# Exploiting microalgal diversity for sterol production

**DOI:** 10.3389/fpls.2025.1616863

**Published:** 2025-06-30

**Authors:** Omnia H. Abdelkarim, Rene H. Wijffels, Maria J. Barbosa

**Affiliations:** ^1^ Bioprocess Engineering, AlgaePARC, Wageningen University, Wageningen, Netherlands; ^2^ Department of Botany and Microbiology, Faculty of Science, Tanta University, Tanta, Egypt; ^3^ Faculty of Biosciences and Aquaculture, Nord University, Bodø, Norway

**Keywords:** microalgae, sterols production, environmental conditions, genetic engineering, biotechnological application

## Abstract

Sterols are essential for eukaryotic cell membrane integrity and fluidity, and they demonstrate valuable pharmaceutical and nutraceutical benefits, including anti-inflammatory, antioxidant, anti-cancer, and cholesterol-lowering properties. Traditionally, animal, plant, and microbial fermentation sterols face sustainability, economic, and ethical challenges. Microalgae have emerged as a promising alternative due to their biochemical diversity, rapid growth, and controlled cultivation capabilities. This review explores microalgae’s potential for sterol production, highlighting their advantages, including sustainability and sterol profile diversity, while addressing key challenges of low sterol yields, species-dependent variations, and industrial scalability. We discuss how recent advancements in metabolic engineering, cultivation technologies, and process optimization could enhance sterol production. By integrating innovative biotechnological strategies, microalgae hold the potential to become a viable and sustainable sterol source for pharmaceutical, nutraceutical, and industrial applications.

## Graphical Abstract

Sterols in microalgae: influencing factors, challenges, solutions, and applications

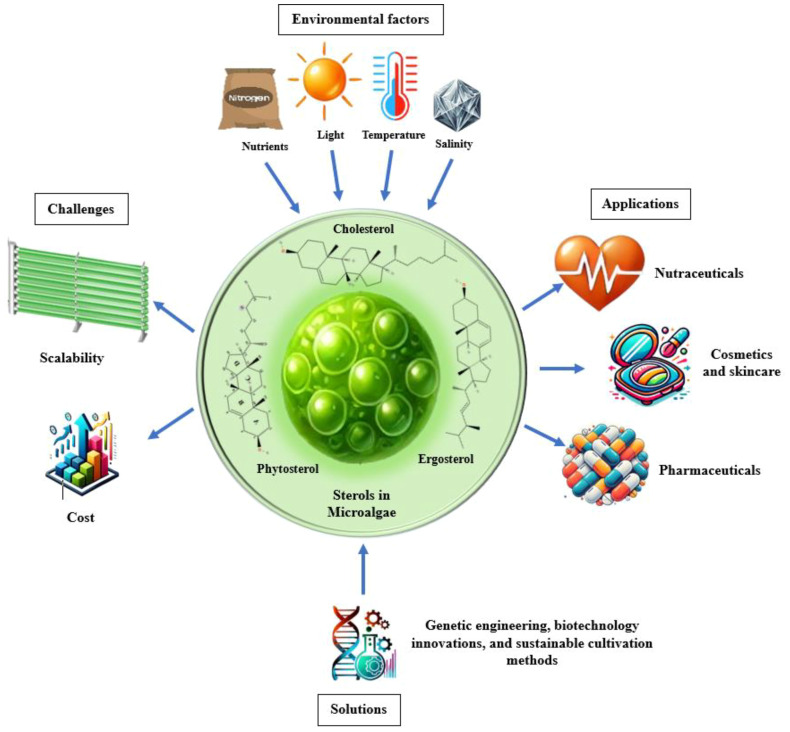

## Introduction

**Figure 1:**
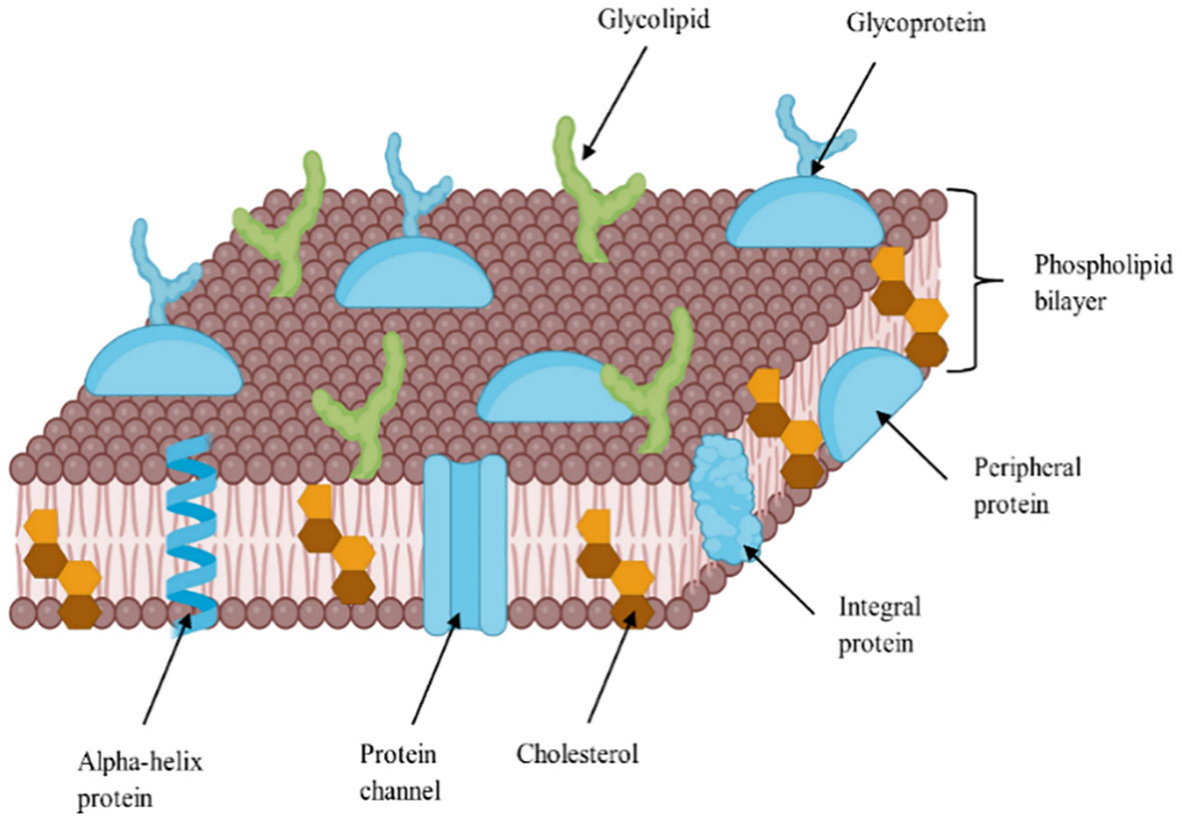
A detailed diagram showing sterol distribution in the lipid bilayer of cell membrane, adapted from (Bodenes, 2017).

**Figure 2:**
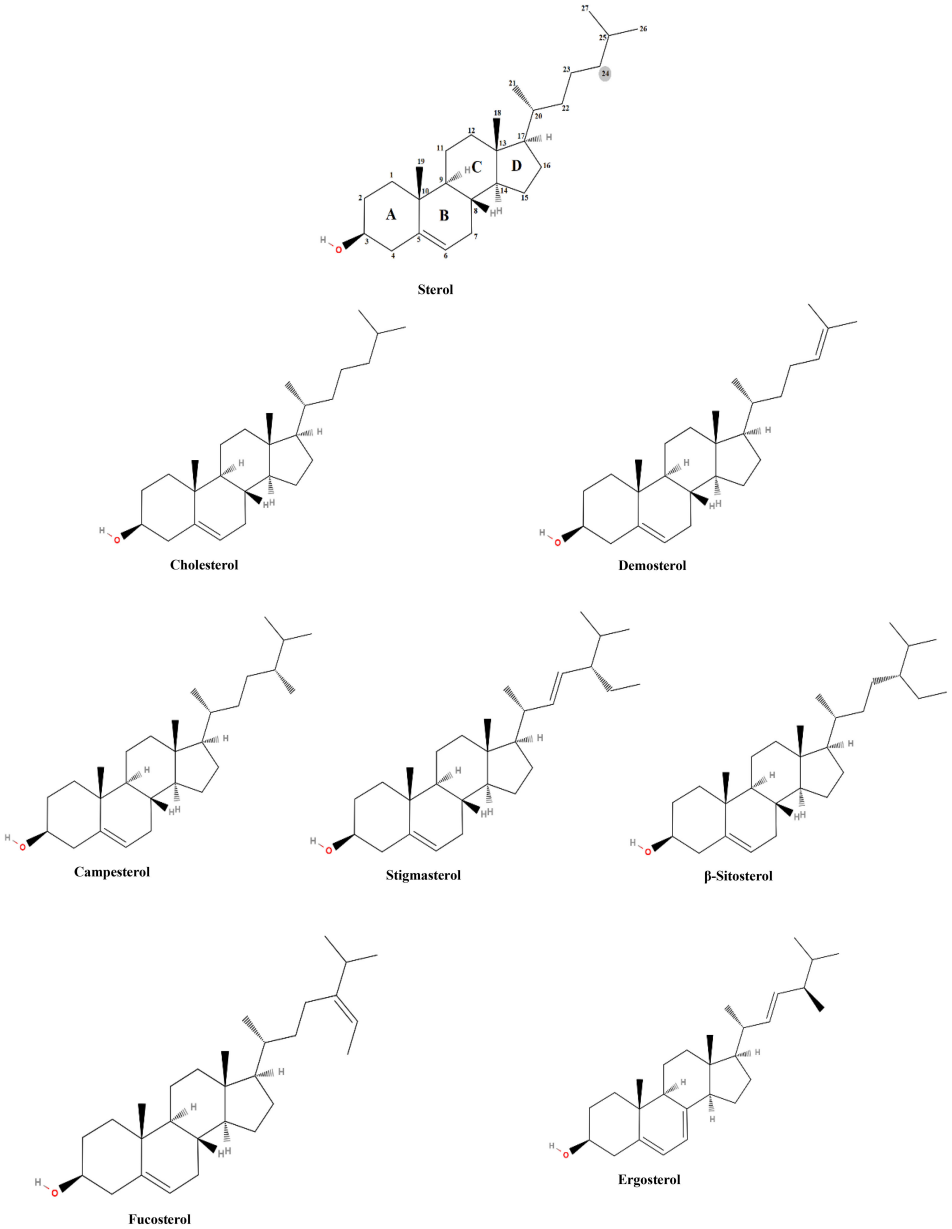
Basic sterol structure and the chemical structure of the most common sterols.

Sterols are vital lipid compounds that play critical roles in cellular structure and function, serving as integral components of eukaryotic cell membranes (**Figure 1**). They regulate membrane fluidity and integrity, influence intracellular signaling, and modulate enzyme and receptor activities, impacting fundamental biological processes such as gene expression, cell division, and apoptosis. Additionally, sterols are key intermediates in isoprenoid lipid biosynthesis, a pathway essential for synthesizing bioactive molecules involved in cellular homeostasis (Volkman, 2003). Structurally, sterols are characterized by a tetracyclic cyclopenta[α]phenanthrene ring system, which consists of three cyclohexane rings (A, B, and C) and one cyclopentane ring (D) (**Figure 2**). Their amphipathic nature, attributed to the presence of both hydrophilic and hydrophobic regions, allows them to integrate seamlessly into the phospholipid bilayer, where they regulate membrane properties. The hydroxyl (-OH) group in ring A is particularly significant, forming hydrogen bonds and other key molecular interactions and stabilizing membrane architecture. The B ring contributes to sterol planarity, while the C ring influences side-chain orientation at C20, which affects sterol function. The D ring and its bonded side chains further define sterol specificity and interactions, influencing their biochemical roles and structural adaptability (Fagundes et al., 2020). Due to their diverse biological functions, sterols have garnered significant interest in the pharmaceutical, nutraceutical, cosmetic, and food industries. They are extensively used as cholesterol-lowering agents in pharmaceuticals (Poli et al., 2021; Barkas et al., 2023), as functional supplements in nutraceuticals, as anti-aging compounds in cosmetics (Pérez-Sánchez et al., 2018; Bjørklund et al., 2022), and as stabilizers (Mohammadi et al., 2020) and bioactive ingredients in food formulations (T.B.D.A, 2024; Association, N.L, 2024). Given their versatility and industrial significance, ongoing research aims to explore alternative, sustainable sources of sterols to address growing market demand while ensuring environmental and economic feasibility.

## Common sterols, commercial sources of sterols, and their applications

The production of sterols, vital for applications in food, healthcare, and pharmaceutical industries, relies on diverse feedstocks, including animal, fish, plant, microbial, and synthetic sources, producing distinct sterols such as cholesterol, squalene, phytosterols, and ergosterol (Zio et al., 2024).

Cholesterol, primarily sourced from animal fats and lanolin, is indispensable in the pharmaceutical industry for synthesizing steroid hormones such as corticosteroids, estrogens, and testosterone (Cerqueira et al., 2016). These are essential for regulating metabolism, immune and nervous responses, reproductive functions, and various therapies, including hormonal replacement and anti-inflammatory treatments. Cholesterol is also critical in manufacturing myelin sheaths and vitamin D (Cruz et al., 2013; Hofmaenner et al., 2022). Moreover, cholesterol also plays a role in bile acid synthesis, which is crucial for metabolic processes and utilized in the food industry to enhance the texture of dairy-based desserts and baking fats (Staels and Fonseca, 2009). Additionally, its emollient properties make it valuable in cosmetics for skin care products, and it is a fundamental component for lipid bilayer studies in research and diagnostics (Volkman, 2003).

Phytosterols (brassicasterol, stigmasterol, campesterol, and beta-sitosterol), similar in structure and function to cholesterol, are plant-derived and extracted from vegetable oils, tall oil, legumes, nuts, seeds, whole grains, and dried fruits (Fernandes and Cabral, 2007). and rice bran oil (Davis and Dean, 2016; Sujith Kumar et al., 2017). Known for reducing serum cholesterol levels by competing with dietary cholesterol for absorption in the gastrointestinal tract, phytosterols are widely used in cholesterol-lowering functional foods and nutritional supplements aimed at cardiovascular health (Poli et al., 2021). They are also valued in cosmetics for their antioxidative, anti-inflammatory, and anticancer properties and their benefits for skin barrier enhancement (Feng et al., 2020).

Squalene, a triterpene hydrocarbon primarily derived from shark liver oil or plants, is used across various industries due to its unique emollient properties. It enhances skin hydration and elasticity without leaving a greasy residue (Fiume et al., 2023). In the pharmaceutical industry, squalene is crucial as an adjuvant in flu and COVID-19 vaccines to enhance immunogenicity (Fox, 2009; Keech et al., 2020). It also finds applications in dietary supplements for its antioxidant benefits and is used in the food industry to inhibit oxidation and extend shelf life (Fiume et al., 2023).

Ergosterol is produced through microbial fermentation, offering a renewable alternative that sidesteps some ethical and sustainability issues associated with animal and plant sources (Papoutsis et al., 2020). It is used to produce vitamin D2 and is in demand primarily by the pharmaceutical and dietary supplement industries (Rangsinth et al., 2023).

Synthetic sterols are produced to meet the needs of various industries, which exceeds what can be feasibly extracted from natural sources (Kumari et al., 2013), and addresses several challenges associated with natural sterol extraction. Synthetic sterols offer several advantages over their natural counterparts. From an economic perspective, synthetic sterols can be produced more cost-effectively, particularly when scaled up to meet industrial demands. In terms of quality, synthetic sterols can be manufactured to have a consistent composition and high purity levels, which is critical for pharmaceutical applications (Feltrin et al., 2020). This consistency ensures that the sterols meet stringent regulatory standards and perform reliably in their intended applications. Moreover, synthetic production can mitigate the environmental impact associated with the large-scale extraction of natural sterols, offering a more sustainable solution (Tata et al., 2015).

## Limitations of commercial sterol sources

The current sources of sterols, whether derived from plants, animals, fungi, or synthesized chemically, face several limitations that can affect their sustainability, economic viability, and overall availability. For instance, the extraction process is often complex and costly due to the low concentrations of sterols in animal tissues (**Table 1**) (Kritchevsky and Chen, 2005; Dinh et al., 2011), which typically range from 0.05-0.3% (w/w) in most tissues, with variations depending on the source (e.g., 0.06-0.1% in beef, 1.0-1.6% in egg yolk, and 2-3% in brain tissue). Animal-derived sterols face health concerns, including the potential for contamination with pathogens or harmful substances. Moreover, ethical challenges related to animal welfare and the environmental implications of animal farming include significant greenhouse gas emissions and resource consumption (Randhir et al., 2020). These factors make animal-derived sterols less sustainable.

**Table 1:** Comparison of sterol content in different biological sources.

Source category	Specific source	Sterol Content (% w w^-1^)	Ref.
Animal Tissue	General	0.05 - 0.3	(Kritchevsky and Chen, 2005; Dinh et al., 2011)
	Beef	0.06 - 0.1	(Kritchevsky and Chen, 2005; Dinh et al., 2011)
	Egg yolk	1.0 - 1.6	(Kritchevsky and Chen, 2005; Dinh et al., 2011)
	Brain tissue	2 - 3	(Kritchevsky and Chen, 2005; Dinh et al., 2011)
Plant sources	Vegetable oils	0.1 - 0.5	(Moreau et al., 2002)
	Cereal grains	0.8- 3	(Piironen et al., 2000)
Fungal sources	Baker’s yeast	0.1 - 2	(He et al., 2007)
	Mushrooms	0.2 - 0.8	(Mattila et al., 2002)
Microalgae	*Pavlova lutheri*	up to 5	(Ahmed et al., 2015)
	General	0.5 - 3	(Volkman, 2003)

While phytosterols are generally more sustainable than animal sources, the scalability of phytosterols is dependent on agricultural factors such as crop yields and weather conditions. The main limitation of phytosterols is their low concentration in most plant sources, ranging from 0.8-3% (w/w) in cereal grains (Moreau et al., 2002) to 0.1-0.5% in vegetable oils (Piironen et al., 2000), which requires processing large amounts of raw material to obtain significant quantities of sterols (Fagundes et al., 2020). This process is resource-intensive and can be environmentally damaging, involving extensive land use, water consumption, and the application of pesticides and fertilizers. The processes required to extract and purify sterols to the high purity required for pharmaceutical and food-grade products are complex and expensive, posing economic challenges (Randhir et al., 2020). Furthermore, the variability in sterol content due to factors like seasonality and geographic location can lead to inconsistent quality and supply. Notably, plants do not produce cholesterol, a critical sterol for vitamin D3 synthesis, highlighting a gap in sterol source capabilities (Sijil et al., 2022).

Fungal sterols, particularly ergosterol, are derived from fungi like yeast and mushrooms. While fungi can be cultivated more sustainably than animals, developing fermentation-based sterols, the extraction and purification processes are still labor-intensive and costly. Additionally, the sterol content in fungi is relatively modest, with concentrations ranging from 0.1-2% (w/w) in baker’s yeast (He et al., 2007) and 0.2-0.8% in mushrooms (Mattila et al., 2002), which is comparable to microalgal sterol content. However, unlike microalgae, fungi cannot produce cholesterol, which limits their versatility as a sterol source. Additionally, there are also challenges related to maintaining sterile growth conditions and preventing contamination, which can complicate production and increase costs (Zhabinskii et al., 2022).

Synthetic sterols, while offering consistency and scalability, come with their own set of limitations. The production process is energy-intensive and relies heavily on petrochemical inputs, leading to a significant environmental footprint (Feng et al., 2020). Synthetic production can also be costly due to the complexity of chemical processes and the price of raw materials. Furthermore, consumer preference often leans towards natural products, posing a marketability challenge for synthetic sterols. Potential contaminants from chemical synthesis and stringent regulatory requirements further complicate the production and application of synthetic sterols (Tata et al., 2015).

## Microalgae as an alternative sterol source

The limitations of commercial sterol sources have catalyzed research and development efforts to discover more sustainable, cost-effective, and socially acceptable alternatives. Microalgae stands out as a promising solution, offering numerous advantages regarding sustainability, efficiency, and biochemical diversity. While the sterol content in most microalgae species is also relatively low compared to some commercial sources, typically ranging from 0.5-3% (w w^-1^) of dry biomass (Volkman, 2003) (**Table 1**), particular species such as *Pavlova lutheri* achieving concentrations up to 5% (w w^-1^) (Ahmed et al., 2015) under optimized conditions, making them competitive with traditional sources. Additionally, microalgae produce a diverse range of sterols, including cholesterol and ergosterol, which plants cannot produce, providing a broad spectrum of bioactive molecules that can be tailored for specific applications (Sijil et al., 2022). Additionally, the ability to cultivate microalgae in controlled environments ensures consistent sterol production, mitigating the seasonal and environmental fluctuations observed in plant-derived sterols.

Beyond sterol content, microalgae present distinct advantages in scalability and environmental sustainability. Unlike plant sources, microalgae can be cultivated on non-arable land and in saline or brackish water, reducing competition with food crops (Abdelkarim et al., 2024). Additionally, microalgae can capture CO_2_ from industrial emissions, contributing to carbon reduction efforts. This starkly contrasts the environmental impact of animal farming, intensive agriculture, petrochemical-based synthetic production, and microbial fermentation, which often relies on agricultural feedstocks. Furthermore, microalgae exhibit rapid growth rates and high biomass productivity, translating to more efficient production processes (Abo-Shady et al., 2023). Under optimal conditions, some species can double their biomass within hours, leading to higher sterol yields in a shorter time frame than traditional sources, which require lengthy cultivation periods and extensive resource inputs (Gheda et al., 2021; Abo-Shady et al., 2023).

A significant advantage of microalgal cultivation is the ability to precisely control environmental parameters in closed systems, such as photobioreactors. These systems regulate light, temperature, pH, and nutrient supply, ensuring consistent sterol production with minimal contamination risks (Fagundes et al., 2020; Randhir et al., 2020). However, while laboratory-scale cultivation has demonstrated high sterol yield and quality, scaling up these controlled environments to industrial levels presents significant challenges. The high setup and operational costs of photobioreactors remain a barrier to commercialization. Nevertheless, advances in process optimization, photobioreactor engineering, and cost-effective nutrient sourcing steadily improve commercial feasibility (Wijffels et al., 2013).

Microalgae offer a sustainable and biochemically diverse alternative to traditional sterol sources, with advantages including rapid growth, environmental adaptability, and potential for controlled production. However, scaling up remains an ongoing challenge, requiring advancements in bioprocess engineering, metabolic optimization, and cost reduction strategies. If these barriers can be overcome, microalgae have the potential to become a high-yield, industrially viable source of sterols for nutraceutical, pharmaceutical, and green biotechnology applications.

The sterol composition across microalgae species displays remarkable diversity, reflecting complex biosynthetic pathways and environmental adaptation (**Table 2**). It is important to note that this review excludes dinoflagellates, a group known for producing highly unusual sterols such as dinosterol and its derivatives. Due to the complexity and variability of their metabolic networks, dinoflagellates warrant a separate and more detailed examination. Their metabolic plasticity and capacity for hybrid biosynthetic routes challenge current models of sterol evolution and remain an open area for future investigation.

**Table 2:** Sterol profiles (% of total sterol) and total sterol (mg/g_dw_) in various microalgae species across different phyla.

Phylum	Microalgae	The dominant sterols	Ref.
Cyanobacteria	*Nostoc commune*	Campesterol (35.2%), β-sitosterol (28.7%), and clionasterol (24.3%), cholesterol (7.4%)	(Kohlhase and Pohl, 1988; Rasmussen et al., 2008)
	*Spirulina platensis*	Cholesterol (9.3%), campesterol (21.8%), stigmasterol (15.7%), β-sitosterol (53.2%)	(Ramadan et al., 2008)
	*Phormidium autumnale*	Squalene (0.426mg/g), cholesterol (0.821mg/g), stigmasterol (0.455mg/g), β-sitosterol(0.279mg/g)	(Fagundes et al., 2019)
	*Anabaena* sp	Cholesterol (~7.8%), brassicasterol (~1%), campesterol (1.6%), stigmasterol or poriferasterol (20.7%), sitosterol or clionasterol (28.9%), stigmastanol (40.2%)	(Kohlhase and Pohl, 1988; Hai et al., 1996)
	*Spirula maxima*	Cholesterol (8.5%), Cholestan-3B-ol (1.2%), 24-Ethylcholest-5-ene-3B-ol (sitosterol or clionasterol) (79.5%), 24Ethylcholestan-38-01 (stigmastanol) (1.9%), 24-Ethylcholesta-7,22-diene-3/?-ol (chondrillasterol) (1.5%), 24-Ethylcholesta-7-ene-3/3-ol (22-dihydrochonodrillasterol) (1.8%)	(Rzama et al., 1994)
Chlorophyta	*Chlorella* sp	Cholesterol (4.9%), Ergosterol (55.9%), 7-Dehydroporiferasterol (17.3%), 9(11)-Dehydroergosterol (5.2%), 7,9(11)-Bisdehydroporiferasterol (3.6%)	(Yasukawa et al., 1996)
	*Chlorella vulgaris*	Ergosterol (~50%) and fungisterol (~26%)	(Martin-Creuzburg and Merkel, 2016)
	*Chlorella luteoviridis*	Poriferasterol (~70%), 22-dihydrobrassicasterol (~20%), and clionasterol (~5%)	(Martin-Creuzburg and Merkel, 2016)
	*Choricystis minor*	Ergosterol (~58%), fungisterol (~15%), 22-dihydrochondrillasterol (~10%), and lichesterol (~10%)	(Martin-Creuzburg and Merkel, 2016)
	*Scenedesmus quadricauda*	Fungisterol, chondrillasterol and 22-dihydrochondrillasterol	(Piepho et al., 2010)
	*Chlamydomonas globosa*	Fungisterol and ergosterol	(Piepho et al., 2010)
	*Chlamydomonas reinhardtii*	Cycloart-24(25)-enol (0.3%), Ergosta-8,25(27)-dienol (2.7%), Ergosta-7,25(27)-dienol (2.6%), Porifersta-8,25(27)-dienol(1.6%), Ergost-7-enol (3.5%), Porifersta-7,25(27)-dienol (0.5%), Poriferst-7-enol (0.9%), Ergosta-5,7,22-trienol (50.8%), and Porifersta-5,7,22-trienol (37.2%)	(Miller et al., 2012)
	*Chlamydomonas reinhardtii*	Ergosterol (~50%), 7-dehydroporiferasterol (~38%), and fungisterol (~10%)	(Martin-Creuzburg and Merkel, 2016)
	*Chlamydomonas globosa*	Ergosterol (~70%), 7-dehydroporiferasterol (~19%), and 5-dihydroergosterol (~7%)	(Martin-Creuzburg and Merkel, 2016)
	*Scenedesmus obliquus*	Chondrillasterol (~50%), fungisterol (~35%) and 22-dihydrochondrillasterol (~10%)	(Martin-Creuzburg and Merkel, 2016)
	*Scenedesmus quadricauda*	Chondrillasterol (~57%), fungisterol (~22%) and 22-dihydrochondrillasterol (~20%)	(Martin-Creuzburg and Merkel, 2016)
	*Ankistrodesmus fusiformis*	Chondrillasterol (~25%), 22 dihydrochondrillasterol (~25%), fungisterol (~22%) and 24-methylenelathosterol (~18%)	(Martin-Creuzburg and Merkel, 2016)
	*Ankistrodesmus falcatus*	22-dihydrochondrillasterol (~55%) and fungisterol (~38%)	(Martin-Creuzburg and Merkel, 2016)
	*Monoraphidium obtusum*	22-dihydrochondrillasterol (~38%), fungisterol (~38%) and chondrillasterol (~20%)	(Martin-Creuzburg and Merkel, 2016)
	*Monoraphidium minutum*	22-dihydrochondrillasterol, fungisterol, 24β-ethylcholesta-7,25(27)-dien-3β-ol and chondrillasterol (~21%)	(Martin-Creuzburg and Merkel, 2016)
	*Dunaliella tertiolecta*	7-dehydroporiferasterol (5.178 mg/g_dw_), ergosterol (2.935 mg/g_dw_), fungisterol (1.492 mg/g_dw_), 22-dihydrochondrillasterol (0.784 mg/g_dw_), chondrillasterol (0.374 mg/g_dw_), and 5-dihydroergosterol (0.214 mg/g_dw_)	(Francavilla et al., 2010)
	*Dunaliella salina*	7-dehydroporiferasterol (3.863 mg/g_dw_), ergosterol (2.006 mg/g_dw_), fungisterol (1.260 mg/g_dw_), 22-dihydrochondrillasterol (0.593 mg/g_dw_), chondrillasterol (0.579 mg/g_dw_), and 5-dihydroergosterol (0.184 mg/g_dw_)	(Francavilla et al., 2010)
	*Dunaliella tertiolecta*	Ergosterol and 7-dehydroporiferasterol	(Caroprese et al., 2012)
	*Pyramimonas cordata*	Stigmasterol	(Ponomarenko et al., 2004)
	*Dunaliella salina*	Cholesterol (5.2%), Ergosterol (28.32%), 24-Methylcholesta-7,22-dienol (1.14%), Fungisterol (4.05%), 24-Ethylcholesta-5,7,22-trienol (58.5%), Chondrillasterol (0.64%), Dihydrochondrillasterol (2.1%)	(Ahmed et al., 2015)
	*Tetraselmis sp. M8*	4.32 mg/g_dw_ Cholesterol (0.34%), Ergost-5-enol (99.66%)	(Ahmed et al., 2015)
	*Tetraselmis chui*	Cholesterol (0.15%), Ergost-5-enol (99.85%)	(Ahmed et al., 2015)
	*Tetraselmis suecica*	Cholesterol (0.23%), Ergost-5-enol (99.77%)	(Ahmed et al., 2015)
Haptista	*Isochrysis galbana*	24-Oxocholesterol acetate, Ergost-5-en-3β-ol, Cholest-5-en-24-1,3-(acetyloxy)-,3β-ol, and sitosterol	(Prakash et al., 2010)
	*Isochrysis galbana*	Cholesterol (1.76%), Epibrassicasterol (98.24%), Fucosterol (29.1%)	(Ahmed et al., 2015)
	*Pavlova lutheri*	26.05 mg/g_dw_ Epibrassicasterol (0.53%), Ergost-5-enol (8.36%), Epicampestanol (0.39%), Poriferasterol(35.61%), 24-Ethylcholest-22-enol (4.31%), Clinasterol (24.26%), 4-Alpha-methylergost-22-enol (2.05%), 4-Alpha-methylporiferast-22-enol (22%), 22-Dehydroethylpavlovol (0.42%), Methylpavlovol (1.98%)	(Ahmed et al., 2015)
	*Pavlova salina*	0.16 mg/g_dw_ Ergosterol (1.43%), Ergost-5-enol (9.85%), Poriferasterol(35.2%), 24-Ethylcholesta-5,7,22-trienol (3.6%), Clinasterol (24.62%), 4-Alpha-methylporiferast-22-enol (15.74%)	(Ahmed et al., 2015)
Rhodophyta	*Porphyridium cruentum*	Cholesterol, campesterol, stigmasterol, and sitosterol.	(Durmaz et al., 2007)
	*Rhodosorus* sp.CS-210	4,24-Dimethyl-5a-cholest-7-en-3β-ol (5.8%), 4-Methyl-5a-cholesta-7,22-dien-3βol (12.3%), 24-Methyl-5α-cholest-7-en-3β-ol (3.4%), 24-Methyl-5α-cholesta-7,22E-dien-3β-ol (2.2%), 24-Methylcholest-5-en-3β-ol (Campesterol) (2.5%), and 24-Methylcholesta-5,22E-dien-3β-ol (71.8%)	(Dunstan et al., 2005)
Eustigmatophyte	*Nannochloropsis* sp BR2	4.04 mg/g_dw_ Cholesterol (77.73%), Fucosterol (8.28%), and Isofucosterol (13.98%)	(Ahmed et al., 2015)
	*Nannochloropsis oceanica*	Cholesterol, fucosterol, isofucosterol, and 24-Methyl elencilesterol	(Lu et al., 2014)
	*Nannochloropsis limnetica*	Cholesterol (~75%), isofucosterol (~15%), and sito-/clionasterol (~10%)	(Martin-Creuzburg and Merkel, 2016)
Bacillariophyta (diatoms)	*Cyclotella meneghiniana*	24-Methylenecholesterol (~88%), and 22-dihydrobrassicasterol (~18%)	(Martin-Creuzburg and Merkel, 2016)
	*Stephanodiscus hantzschii*	24-methylenecholesterol (~85%) and desmosterol (~10%)	(Martin-Creuzburg and Merkel, 2016)
	*Gomphonema parvulum*	Epibrassicasterol (~90%) and 5-dehydrostellasterol/ergosterol (~10%)	(Martin-Creuzburg and Merkel, 2016)
	*Phaeodactylum tricornutum*	(24S)-24-Methylcholesta-5,22E-dien-3β-ol (Brassicasterol or Epibrassicasterol)	(Fábregas et al., 1997)
	*Phaeodactylum tricornutum*	Cholesterol (2.71%), Epibrassicasterol (95.34%), Ergost-5-enol (1.94%)	(Ahmed et al., 2015)
	*Nitzschia closterium*	Cholesta-5,24-dien-3β-ol, and 24-Methylcholesta-5,22E-dien-3β-ol	(Barrett et al., 1995)
Cryptophyte	*Rhodomonas* sp. CS-215	(24S)-24-Methylcholesta-5,22E-dien-3β-ol (98.7%), and Cholest-5-en-3β-ol (cholesterol) (1.3%)	(Dunstan et al., 2005)
	*Rhodomonas* sp. CS-694	24-Methylcholesta-5,7,22-trien-3β-ol (Ergosterol) (7.5%), (24S)-24-Methylcholesta-5,22E-dien-3β-ol (91.5%), and Cholest-5-en-3β-ol (1%)	(Dunstan et al., 2005)
	*Proteomonas sulcata*	(24S)-24-Methylcholesta-5,22E-dien-3β-ol (97.3%), and Cholest-5-en-3β-ol (2.7%)	(Dunstan et al., 2005)
	*Rhodomonas salina* CS-174	(24S)-24-Methylcholesta-5,22E-dien-3β-ol (98.9%), and Cholest-5-en-3β-ol (1%)	(Dunstan et al., 2005)
	*Rhodomonas salina* CS-24	(24S)-24-Methylcholesta-5,22E-dien-3β-ol (98.1%), and Cholest-5-en-3β-ol (1.9%)	(Dunstan et al., 2005)
	*Rhodomonas maculata* CS-85	(24S)-24-Methylcholesta-5,22E-dien-3β-ol (82.3%), and Cholest-5-en-3β-ol (17.7%)	(Dunstan et al., 2005)
	*Chroomonas placoidea* CS-200	(24S)-24-Methylcholesta-5,22E-dien-3β-ol (62.5%), Cholest-5-en-3β-ol (2%), and 24-Ethylcholesta-5,22E-dien-3βol (35.5%)	(Dunstan et al., 2005)
	*Cryptomonas ovata*	Stigmasterol and brassicasterol	(Piepho et al., 2010)
	*Cyanophora paradoxa*	24-Methylcholest-5-en-3β-ol (19.4%), 24-Ethylcholest-5-en-3β-ol (β-Sitosterol or Clionasterol) (75.1%), and 24-Ethylcholesta-5,22E-dien-3β-ol (Stigmasterol) (5.5%)	(Leblond et al., 2011)
	*Cryptomonas* sp.	Epibrassicasterol (~55%) and stigmasterol (~45%)	(Martin-Creuzburg and Merkel, 2016)
	*Cryptomonas erosa*	Epibrassicasterol and stigmasterol (~50%)	(Martin-Creuzburg and Merkel, 2016)

The case of cyanobacteria also presents an ongoing scientific debate. Although numerous studies have reported sterol-like compounds in genera such as *Nostoc*, *Anabaena*, *Spirulina*, and *Phormidium*, compelling evidence suggests these may not be synthesized *de novo*. Volkman (2003; 2005) has argued that the sterols detected in cyanobacterial samples, typically in trace amounts below 0.03% of cell dry weight, could originate from external contamination, particularly by eukaryotic organisms such as fungi or yeasts (Hai et al., 1996). This interpretation is supported by studies demonstrating that cyanobacterial cultures become sterol-free when treated with cycloheximide, an inhibitor that selectively suppresses eukaryotic contaminants (Summons et al., 2001). Furthermore, genomic analyses reinforce the scepticism, while some cyanobacterial genomes contain genes with weak homology to sterol biosynthesis enzymes, they universally lack oxidosqualene cyclase (OSC), a critical enzyme that initiates the cyclization of 2,3-oxidosqualene into lanosterol or cycloartenol (Volkman, 2003; 2005). The absence of this key gene suggests that recognized sterol synthesis pathways are not native to cyanobacteria. This has significant implications for interpreting sterol biomarkers in ancient sedimentary rocks. If cyanobacteria are confirmed not to produce C-24 alkylated sterols, their presence in ancient geological records may serve as more robust evidence for early eukaryotic life, rather than ambiguous biosignatures subject to bacterial origin (Cavalier-Smith, 2002a; 2002b).

Among green algae (Chlorophyta), sterol profiles exhibit considerable biochemical diversity, underscoring this group’s evolutionary and ecological plasticity. Additionally, the lack of a universally dominant sterol across Chlorophyta orders and families and the range of compounds detected, such as ergosterol, fungisterol, and unique compounds like 7-dehydroporiferasterol, suggests distinctive cellular processes and adaptation mechanisms. The presence of ergosterol, commonly associated with fungi, in species like *Chlorella* and *Scenedesmus*, assumptions about the taxonomic exclusivity of specific sterols, underscores the conservation of certain biosynthetic traits across evolutionary boundaries. These findings raise questions about the possible horizontal gene transfer or ancient evolutionary retention of fungal-type sterol pathways in specific algal lineages. Notably, *Dunaliella* species within Chlorophyta further expand this diversity by synthesizing sterol derivatives like ergosterol peroxide, which has known antioxidant properties, highlighting the dual utility of microalgal sterols, not only as structural lipids but also as molecules with potential therapeutic and nutraceutical exploitation. Furthermore, sitosterol has been identified as the primary sterol in marine microalgae species like *Isochrysis galbana*, exemplifying the intra-group variability and ecological specialization of marine green algae (Yasukawa et al., 1996; Ponomarenko et al., 2004; Francavilla et al., 2010; Piepho et al., 2010; Prakash et al., 2010; Chen et al., 2014; Lv et al., 2015; Martin-Creuzburg and Merkel, 2016).

Red microalgae (Rhodophyta) predominantly synthesize C-27 sterols, with cholesterol frequently emerging as the most abundant. *Porphyridium cruentum* displays a sterol composition including cholesterol and stigmasterol, a sterol typically associated with animals and plants, respectively, which raises intriguing questions about evolutionary development. Such overlapping sterol signatures may reflect convergent evolution, where similar selective pressures in membrane structure or signaling led to independent development of comparable biosynthetic pathways, or gene transfer events that transmitted biosynthetic capabilities between distant lineages (Durmaz et al., 2007).

Among heterokontophytes, distinct patterns are evident. The Eustigmatophyceae (Gyrista) species *Nannochloropsis* exhibits a simpler profile with cholesterol and fucosterol (Lu et al., 2014; Ahmed et al., 2015; Martin-Creuzburg and Merkel, 2016). This simplicity may reflect genome streamlining or ecological specialization. In contrast, diatoms, another significant microalgal group, are characterized by a predominance of 24-methylenecholesterol, a compound that is thought to contribute to their membrane rigidity and is likely an adaptation to their siliceous frustules and aquatic habitats (Barrett et al., 1995; Fábregas et al., 1997; Martin-Creuzburg and Merkel, 2016). These differences reinforce the notion that sterol profiles are shaped not only by phylogeny but also by ecological and functional constraints. Cryptophyte species like *Cryptomonas* and *Rhodomonas* further enrich this spectrum with sterols such as epibrassicasterol and brassicasterol, adding to the biochemical repertoire available within microalgae. These compounds may confer selective advantages in specific niches, such as variable salinity or light environments (Piepho et al., 2010; Leblond et al., 2011; Martin-Creuzburg and Merkel, 2016). Their presence underscores the ecological versatility and adaptive potential of cryptophyte species.

The extensive variability in microalgal sterol profiles reveals a sophisticated interplay between genetic inheritance, ecological adaptation, and evolutionary innovation. These diverse biosynthetic capabilities not only provide fundamental insights into algal evolution and taxonomy but also hold immense promise for applied biotechnology. Unique sterols with antioxidant, antifungal, or membrane-modulating properties may be harnessed for use in functional foods, cosmetics, pharmaceuticals, or biomaterials.

## Sterol biosynthesis in different organisms

**Figure 3:**
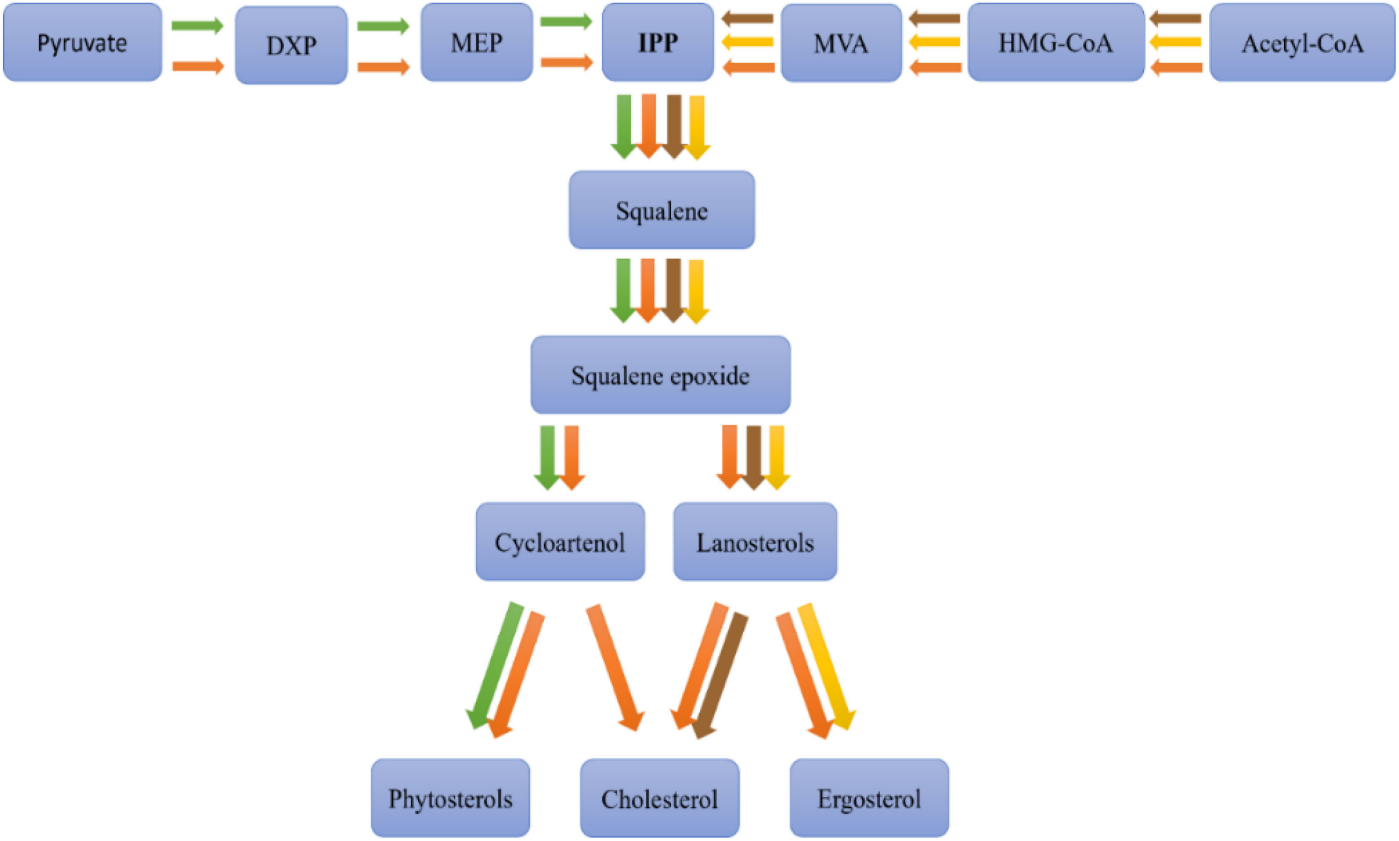
Sterols biosynthetic pathways among different organisms, animal (brown), plant (green), fungi (yellow), and microalgae (orange). Adapted from (Lu et al., 2014), DXP: 1-deoxy-D-xylulose-5-phosphate, MEP: 2-C-methyl-D-erythritol 4-phosphate, IPP: isopentenyl pyrophosphate, MVA: mevalonate, HMG-CoA: 3-hydroxy-3-methylglutaryl coenzyme A, Acetyl-CoA: acetyl coenzyme A.

Sterol biosynthesis involves biochemical pathways that differ among various organisms, such as animals, plants, fungi, and microalgae (**Figure 3**).

In animals, cholesterol biosynthesis primarily occurs in the liver, following the mevalonic acid (MVA) pathway. This pathway initiates with the condensation of acetyl-CoA molecules to form 3-hydroxy-3-methylglutaryl-CoA (HMG-CoA), which is subsequently reduced to mevalonate by HMG-CoA reductase, a pivotal regulatory enzyme in cholesterol synthesis. The conversion of mevalonate through several phosphorylation and decarboxylation steps yields isopentenyl pyrophosphate (IPP), a foundational sterol building block. IPP is transformed into squalene via a sequence of reactions, which then cyclizes to produce lanosterol, the precursor to cholesterol, through multiple enzymatic modifications (Fagundes et al., 2020).

In plants, phytosterol biosynthesis commences similarly from acetyl-CoA, following either an analogous pathway to that in animals or diverging through the methylerythritol phosphate (MEP) pathway from pyruvate to form IPP and subsequently squalene. The plant pathway diverges post-squalene synthesis, with squalene converting to 2,3-oxidosqualene and then to cycloartenol, a critical intermediate. Cycloartenol undergoes various enzymatic transformations to yield phytosterols like sitosterol, stigmasterol, and campesterol (Du et al., 2022).

Fungi synthesize ergosterol, which serves a similar structural role to cholesterol in animals and phytosterols in plants, but follows a distinct biosynthetic pathway. Like other sterol-producing organisms, fungi begin with acetyl-CoA and progress through the mevalonate (MVA) pathway to generate isopentenyl pyrophosphate (IPP) and subsequently squalene. However, post-squalene synthesis, the pathway diverges, leading to lanosterol, which undergoes unique enzymatic modifications to form ergosterol rather than cholesterol (as in animals) or phytosterols (as in plants). These modifications involve specific enzymes that differentiate fungal sterol biosynthesis from other eukaryotic pathways (Singh et al., 2023).

**Figure 4:**
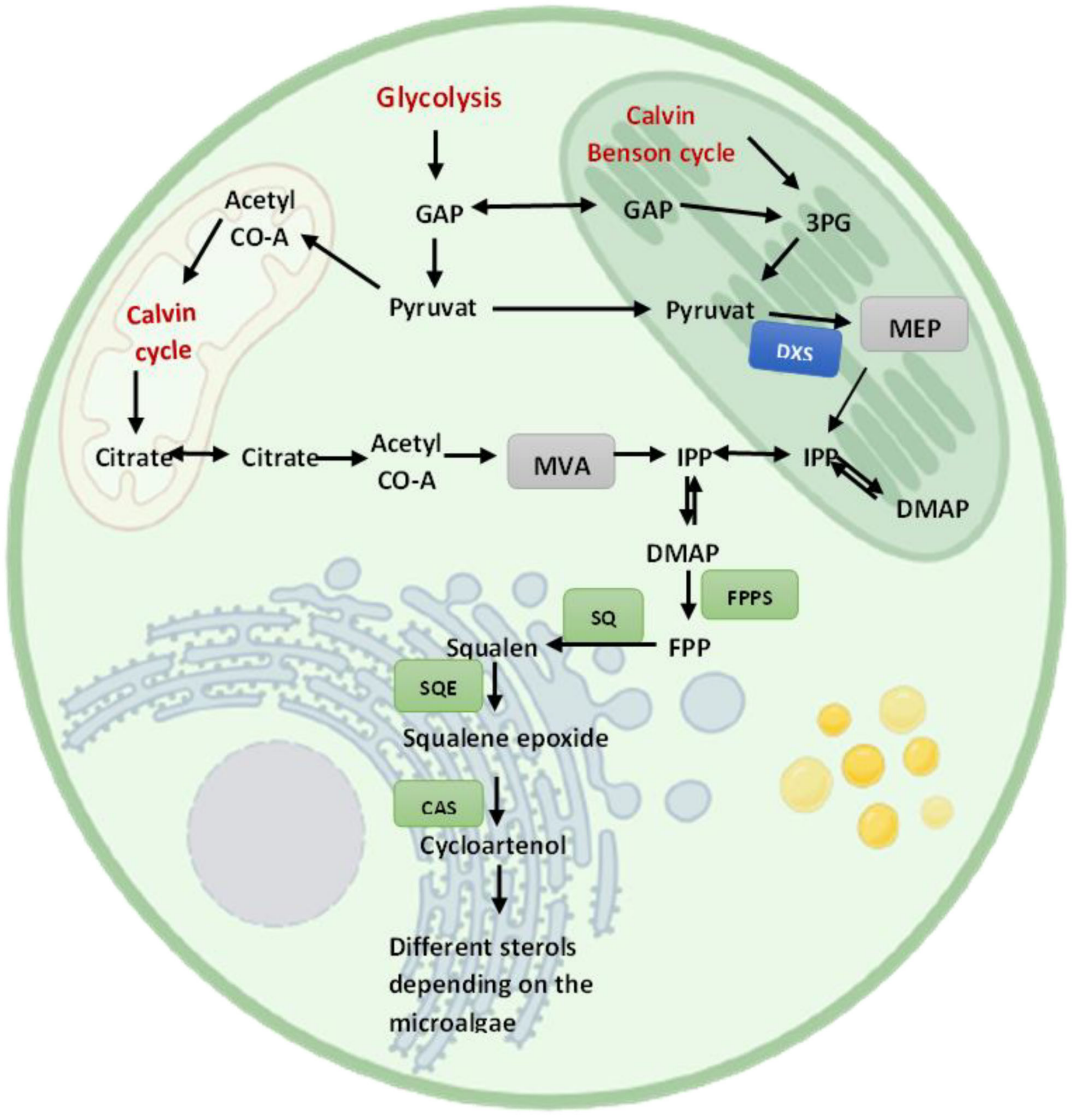
A simplified illustration of sterol biosynthesis pathways in microalgae.

**Figure 5:**
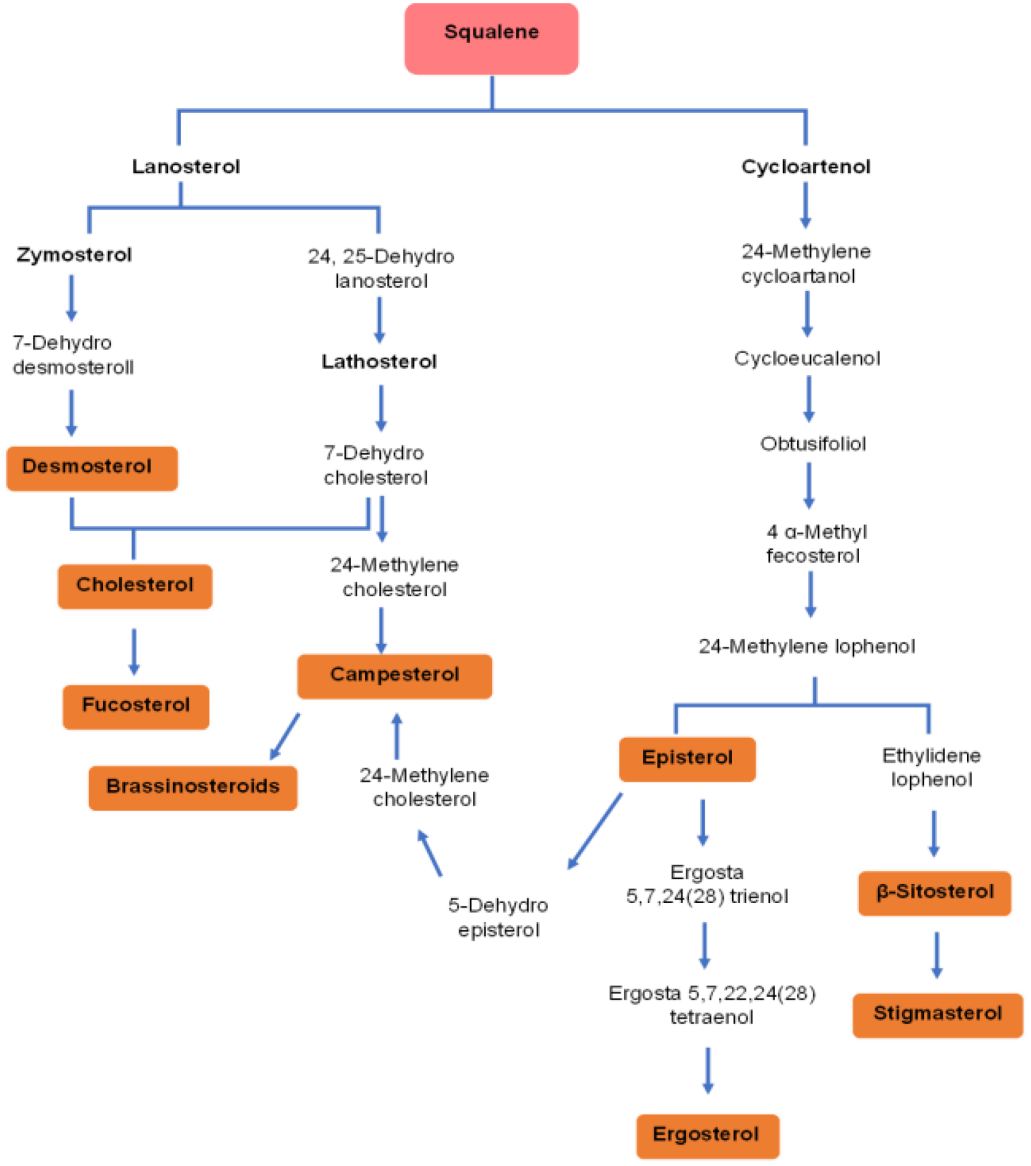
Diversity of sterols derived from squalene in microalgae. This pathway diagram illustrates the complex biosynthetic routes by which microalgae convert squalene into various bioactive sterols, via two primary branches: the lanosterol pathway (common in animals and some microalgae) or the cycloartenol (typical in plants and many microalgae). These pathways demonstrate the biochemical diversity of microalgal sterol synthesis, with end products including cholesterol, fucosterol, campesterol, ergosterol, β-sitosterol, and stigmasterol.

Microalgae exhibit unique and variable sterol biosynthesis pathways (Fagundes and Wagner, 2021), reflecting their evolutionary lineage and the broad diversity of sterols they can produce (**Figure 4**). Sterol biosynthesis in microalgae typically begins with acetyl-CoA, converting to IPP via the mevalonate (MVA) pathway in the cytosol or, alternatively, some species utilize the MEP pathway in chloroplasts (Zhao et al., 2013). Following the synthesis of squalene, a series of enzymatic steps result in the formation of various key intermediates like lanosterol or cycloartenol, leading to a plethora of sterols, including cholesterol, ergosterol, fucosterol, sitosterol, and stigmasterol (Pollier et al., 2019) (**Figure 5**).

Specifically, the biosynthesis of stigmasterol and β-sitosterol involves the conversion of 2,3-oxidosqualene to cycloartenol by cycloartenol synthase (CAS), followed by enzymatic transformations, notably Δ24-sterol methyltransferase (SMT) converting cycloartenol to 24-methylenecycloartenol, leading sequentially to β-sitosterol. Stigmasterol synthesis from β-sitosterol involves desaturation by the cytochrome P450 enzyme CYP710A1 (Griebel and Zeier, 2010).

The cholesterol biosynthesis pathway in microalgae diverges after 2,3-oxidosqualene formation, with subsequent conversions leading to cholesterol (Randhir et al., 2020). For ergosterol, unique pathways, such as in *Chlamydomonas reinhardtii*, start with cycloartenol synthesis, indicating shared pathways with phytosterols (Brumfield et al., 2017).

Additionally, microalgae like *Chlorella vulgaris* can synthesize growth-promoting brassinosteroids from campesterol through complex transformations involving early or later C6-oxidation steps leading to brassinolide (Fagundes et al., 2020).

The regulation of sterol biosynthesis in microalgae is influenced by countless factors, including environmental conditions and developmental stages, underscoring the complexity of these pathways. A detailed understanding of these pathways enhances the biotechnological production of bioactive sterols for pharmaceutical and nutraceutical uses. Advances in genetic and metabolic engineering may further optimize these pathways, increasing sterols’ yield and diversity, thus boosting the commercial potential of microalgae as a source of valuable sterols.

## Effect of culture conditions on the sterol content of microalgae

**Figure 6:**
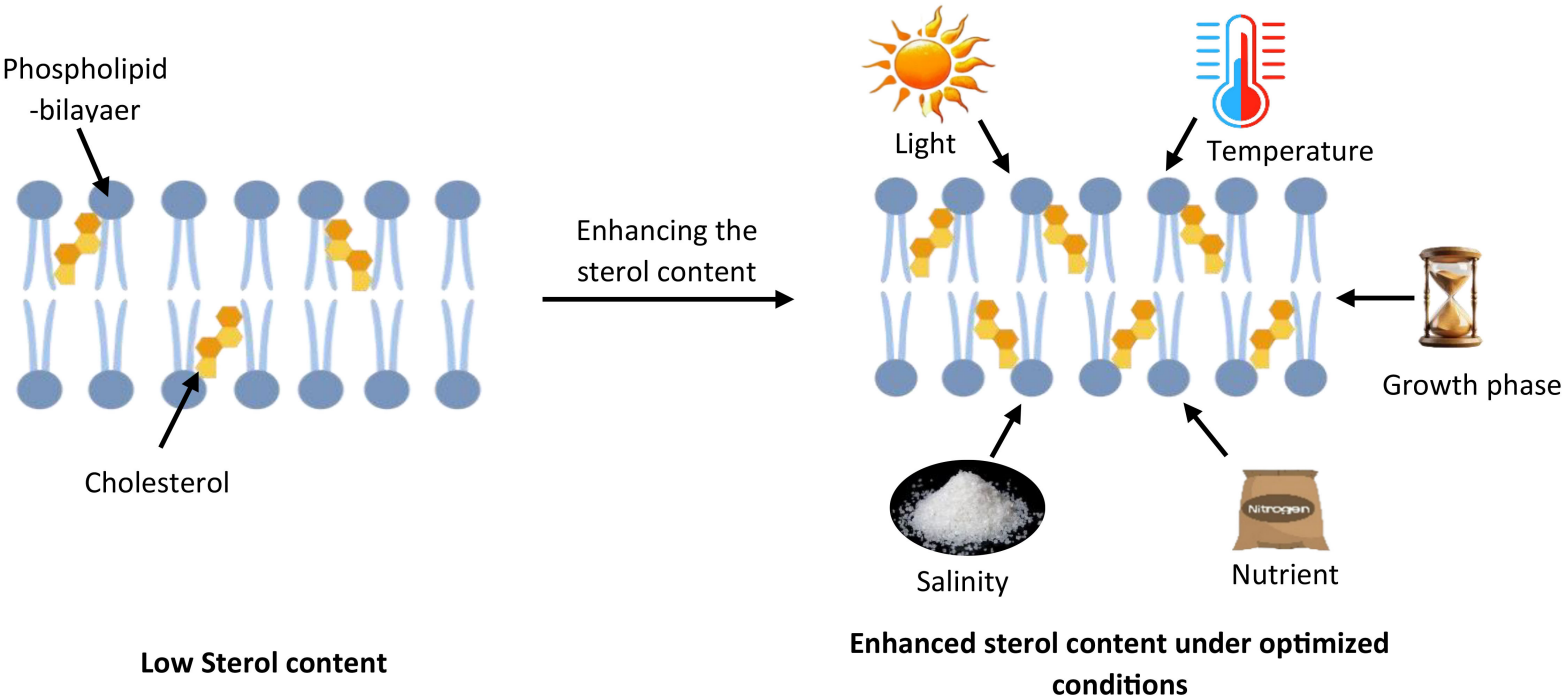
Environmental regulation of sterol composition in microalgal membranes. This schematic depicts how various environmental factors influence sterol content in microalgal cell membranes. The left side shows a phospholipid bilayer with low cholesterol content under standard conditions. The right side demonstrates enhanced cholesterol integration (shown as yellow molecules) within the membrane when environmental conditions are optimized. Key factors that regulate cholesterol biosynthesis and accumulation include light intensity, temperature, salinity, nutrient availability (particularly nitrogen and phosphorus), and growth phase timing.

The sterol content of microalgae is reported to be influenced by several conditions (**Figure 6**), such as light intensity, temperature, nutrient availability, salinity, and growth phases (Lopes et al., 2013) (**Table 3**).

**Table 3:** Influence of cultivation conditions on sterol production in microalgae.

Parameters (Unit)	Microalgal species	Tested conditions (values)	Condition with highest sterol content	Sterols content and type	Ref.
Light intensity μmol_photon_ m^-2^ s^-1^	*Pavlova lutheri*	41.13, 86.83, and 137.1	137.1	Acylated sterol glycosides	(Guedes et al., 2010)
	*Scenedesmus quadricauda*	30, 60, 140, 230, and 490	490	10 mg g^-1^DW Fungisterol, chondrillasterol, and 22-dihydrochondrillasterol	(Piepho et al., 2010)
	*Cyclotella meneghiniana*	30, 60, 140, 230, and 490	490	10 mg g^-1^DW on average 24-methylenecholesterol, and 22-dihydrobrassicasterol	(Piepho et al., 2010)
	*Dunaliella viridis*	35, 250, 700 and 1500	1500	11.1% total lipid	(Gordillo et al., 1998)
	*Nannochloropsis oceanica*	100 and 300	100	14 mg g^-1^DW Cholesterol, Fucosterol, and isofucosterol	(Lu et al., 2014)
	*Haematococcus pluvialis*	90 (16:8 day: night) and 300 (continuous light)	300 (continuous light)	20 mg g^-1^DW Cholesterol, brassicasterol, Δ7-campesterol and sitostanol	(Scodelaro Bilbao et al., 2016)
Temperature °C	*Phaeodactylum tricornutum*	13 and 23°C	13	1.5 mg g^-1^DW Epibrassicasterol, cholesterol	(Véron et al., 1996)
	*Isochrysis galbana*	18-26°C	18	1.644 mg g^-1^DW Stigmasterol	(Durmaz et al., 2008)
	*Scenedesmus quadricauda*	10-25°C	18	Fungisterol 2.5 mg g^-1^DW, chondrillasterol 4.5 mg g^-1^DW, and 22-dihydrochondrillasterol 2.5 mg g^-1^DW	(Piepho et al., 2012)
	*Cyclotella meneghiniana*	10-25°C	10	24-methylenecholesterol 4.5 mg g^-1^DW	(Piepho et al., 2012)
	*Cryptomonas ovata*	10-25°C	———	Brassicasterol 3 mg g^-1^DW at 10°C, and stigmasterol 5 mg g^-1^DW 25°C	(Piepho et al., 2012)
Nitrogen	*Nannochloropsis oceanica*	Nitrogen availability	Nitrogen repletion	11 mg g^-1^DW Cholesterol, Fucosterol, and isofucosterol	(Lu et al., 2014)
	*Thalassiosira weissflogii*	Nitrogen availability	Nitrogen repletion	0.37mg g^-1^DW	(Breteler et al., 2005)
Phosphorus	*Scenedesmus quadricauda*	1, 5, 10, and 50 µM	5 and 10 µM	Fungisterol 5 mg g^-1^DW, chondrillasterol 10 mg g^-1^DW, and 22-dihydrochondrillasterol 5 mg g^-1^DW	(Piepho et al., 2012)
	*Cyclotella meneghiniana*	2.5, 5, 10, and 50 µM	10, and 50 µM	24-methylenecholesterol 5.5 mg g^-1^DW	(Piepho et al., 2012)
	*Cryptomonas ovata*	5, 10, 20, and 50 µM	20 µM	Brassicasterol 7 mg g^-1^DW and stigmasterol 12 mg g^-1^DW	(Piepho et al., 2012)
	*Thalassiosira weissflogii*	Phosphorus availability	Phosphorus repletion	—	(Breteler et al., 2005)
Salinity	*Nitzschia laevis*	10-30g/L	30g L^-1^	5.44 mg g^-1^DW	(Chen et al., 2008)
	*Nannochloropsis salina*	22 to 58 g/L	High NaCl	7.5% of total lipid	(Bartley et al., 2013)
	*Dunaliella salina*, and *tertiolecta*	21–75 g/L	0.6M	Ergosterol 0.89% and 1.3%DW	(Francavilla et al., 2010)
Growth stage	*Porphyridium cruentum*	Growth phases	Stationary phase (20 days)	—	(Durmaz et al., 2007)
	*Nannochloropsis oculata*	Growth phases	Stationary phase	5.3% of total lipid	(Dunstan et al., 1993)
	*Pavlova lutheri*	Growth phases	Stationary phase	5% of total lipid	(Dunstan et al., 1993)
	*Phaeodactylum tricornutum*	Growth phases	Stationary phase	0.87% of total lipid	(Ballantine et al., 1979)
	*Nannochloropsis oceanica*	Growth phases	Late log phase	11.5 mg g^-1^DW Cholesterol, Fucosterol, and isofucosterol	(Lu et al., 2014)

### Light intensity

Recent findings indicate that light intensity significantly affects sterol content in various microalgal species, though comparisons across studies should consider differences in experimental conditions such as biomass concentration, incident light intensity, and optical path length. *Dunaliella viridis* batch-cultured under varying photon flux rates (darkness, 35, 250, 700, and 1500 μmol_photon_ m^−2^ s^−1^ exhibited a marked increase in sterol content with increasing light intensity, particularly at the highest light intensities. The maximum sterol content, 11.1% of total lipid, was achieved at 1500 μmol_photon_ m^−2^ s^−1^, substantially higher than at lower photon fluence rates (1.6% of total lipid in darkness, 3.0% of total lipid at 35 μmol_photon_ m^−2^ s^−1^, 6.5% at 250 μmol_photon_ m^−2^ s^−1,^and 7.2% at 700 μmol_photon_ m^−2^ s^−1^) (Gordillo et al., 1998). Similarly, *Pavlova lutheri* showed increased acylated sterol glycosides with elevated light intensities (Guedes et al., 2010). This pattern was also observed in *Scenedesmus quadricauda* and *Cyclotella meneghiniana*, where higher light intensity increased sterol production (Piepho et al., 2010). The enhancement of sterol levels is hypothesized to be linked with increased photosynthesis activity under high light conditions. This relationship varies among species due to differences in their sterol biosynthetic pathways. Some algae utilize the mevalonate (MVA) pathway, while others rely on the methylerythritol phosphate (MEP) pathway, which is closely associated with chloroplast function. However, certain species have been found to utilize both pathways, integrating intermediates from both metabolic routes to regulate sterol biosynthesis in response to environmental conditions.

Species such as *Haematococcus (H.) pluvialis* and *Nannochloropsis oceanica* exhibit different responses to light intensity. *H. pluvialis* CCALA1081 was batch-cultured under two light regimes: 90 μmol_photon_ m^−2^ s^−1^ with a 16:8 h light/dark photoperiod and 300 μmol_photon_ m^−2^ s^−1^ continuous light. Under a light intensity of 300 μmol_photon_ m^−2^ s^−1^, total phytosterol content increased markedly, reaching approximately 20 mg g_dw_^-1^. However, this overall increase was accompanied by notable shifts in the composition of individual sterols, such as β-sitosterol and 24-methylene cholesterol, which decreased by 25% and 10%, respectively, compared to control conditions at 90 μmol_photon_ m^−2^ s^−1^. Conversely, other sterols such as cholesterol, brassicasterol, Δ7-campesterol, and sitostanol exhibited notable increases by 20%, 10%, 8%, and 5%, respectively, suggesting that elevated light triggers selective adjustment in sterol composition, potentially reflecting adaptive modifications in membrane structure and function (Scodelaro Bilbao et al., 2016). On the other hand, *N. oceanica* cultures were grown at standard light intensity 50 μmol_photon_ m^−2^ s^−1^ and then exposed to two specific light conditions, 100 and 300 μmol_photon_ m^−2^ s^−1^, which demonstrated a decrease in sterol content under high light (300 μmol_photon_ m^−2^ s^−1^) with the reduction primarily in cholesterol levels. However, some phytosterols, such as fucosterol and isofucosterol, increased in concentration under high light, indicating a complex response where overall sterol biosynthesis is repressed, yet specific sterols are selectively increased (Lu et al., 2014). These variations underscore the complexity of light’s impact on sterol metabolism in microalgae, which appears to be species-specific and possibly related to their distinct biosynthetic pathways. While these studies provide valuable insights, the variation in experimental conditions (culture conditions and mode, biomass concentrations, and optical path lengths) makes direct quantitative comparisons challenging. Consequently, further research involving diverse microalgal strains with standardized cultivation conditions is essential to fully understand sterol metabolism and its relationship with light intensity in these organisms.

### Temperature

Temperature influences microalgae’s physiological and metabolic processes, impacting their growth rate, cell size, biochemical composition, and nutrient uptake capabilities. It plays a crucial role in regulating sterol metabolism, where the synthesis of ethyl groups branched at the C24 position serves as a cellular defense mechanism against thermal stress, as elucidated by Beck et al. (2007). This adaptation underscores the potential of manipulating temperature conditions to enhance the biotechnological production of valuable compounds such as squalene.

Further research into the temperature’s impact on sterol content across various microalgal species reveals nuanced responses. For instance, in *Phaeodactylum tricornutum*, sterol content decreased with increasing culture temperature from 13°C to 23°C, with the highest sterol content at 13°C, 1.5 mg gdw^-1^. Similarly, *Isochrysis galbana* demonstrated elevated sterol levels with 1.6 mg g_dw_^-1^ at lower temperatures (18°C compared to 26°C), indicating a possible thermal inhibition of sterol synthesis at higher temperatures (Durmaz et al., 2008).

Conversely, other studies have reported an opposite trend, where sterol production increases with rising temperatures. Investigations by Piepho et al. (2012) into *Scenedesmus quadricauda, Cyclotella meneghiniana*, and *Cryptomonas ovata* revealed that, while sterol content increased at 25°C compared to 10°C in *S. quadricauda* and *C. meneghiniana*, it remained unaffected in *C. ovata’s*. A similar trend was observed in *Botryococcus braunii*, where sterol levels were highest at 25°C but declined at both lower (18°C) and higher (32°C) temperatures (Kalacheva et al., 2002). These findings underscore the complexity of temperature effects on microalgal sterol biosynthesis, suggesting a species-specific and possibly strain-specific response that could be leveraged for targeted biotechnological applications.

### Nutrient availability

Nutrient availability plays a critical role in modulating the biosynthesis and accumulation of sterols in microalgae, acting as a crucial environmental factor influencing lipid metabolism pathways. The relationship between nutrient supply, particularly nitrogen and phosphorus, and sterol content in microalgae is complex and varies across different species, reflecting their adaptive responses to nutrient stress conditions.

#### Nitrogen availability

Nitrogen starvation in microalgae leads to decreased sterol production primarily due to a reallocation of metabolic resources towards essential survival processes. For instance, several species like *Ankistrodesmus falcatus* (Kilham et al., 1997), *Nannochloropsis oceanica* (Lu et al., 2014), *Eudorina unicocca*, and *Volvox aureus* (Zhang et al., 2009) demonstrated a decrease in sterol content under nitrogen limitation. The reason is that under nitrogen deficiency, microalgae prioritize nitrogen conservation for critical functions such as protein synthesis and photosynthesis, diverting resources away from non-essential metabolite synthesis, including sterols. This metabolic shift also enhances the accumulation of energy-storage lipids, such as triacylglycerols (TAGs), competing with sterol biosynthesis for common precursors like acetyl-CoA. Moreover, nitrogen stress may affect the enzymatic activity within the sterol biosynthesis pathway and alter cell growth, reducing sterol production as the demand for membrane components decreases (Lu et al., 2014). This adaptation reflects the organism’s strategic response to nutrient limitation, emphasizing the importance of understanding metabolic regulation for optimizing microalgae-based production systems.

Contrarily, nitrogen limitation was found to elevate free sterol levels in *Botryococcus braunii*, while sterol ester content remained unchanged (Zhila et al., 2005). That is because, under conditions of nitrogen deficiency, microalgae undergo a metabolic shift from synthesizing membrane lipids to accumulating storage lipids, predominantly triacylglycerides (TAGs). This transition is a survival strategy that impacts cell growth and can increase cell volume, as observed in different microalgae species. The enlargement of cell volume during nitrogen starvation has been associated with an enhancement in sterol content attributed to the expansion of the cell membrane (Randhir et al., 2020). These observations underscore the nuanced relationship between nitrogen availability and sterol biosynthesis, indicating that nitrogen stress can either promote or inhibit sterol accumulation depending on the species and the specific metabolic adaptations invoked.

#### Phosphorus availability

The impact of phosphorus on sterol content in microalgae complements the intricate dynamics between nutrient availability and lipid metabolism. Phosphorus, a critical nutrient for algal growth, influences various metabolic processes, including nucleic acids and ATP synthesis. Its availability directly affects the production of sterols, components vital for maintaining cellular membrane integrity and fluidity. An adequate supply of phosphorus can enhance sterol biosynthesis, as evidenced by the increase in sterol content in *Thalassiosira weissflogii* (Breteler et al., 2005)*, Scenedesmus quadricauda* and *Cyclotella meneghiniana* (Piepho et al., 2012) with elevated phosphorus levels. This enhancement is likely due to the role of phosphorus in supporting the overall metabolic activity, including the pathways involved in sterol synthesis. However, the response to phosphorus supplementation can vary among microalgae species, indicating species-specific metabolic strategies for managing nutrient stress. While some species exhibit increased sterol production with higher phosphorus availability, others show no significant change, such as *Cryptomonas ovata*, suggesting that the regulatory mechanisms governing sterol biosynthesis in response to phosphorus availability are complex and multifaceted (Piepho et al., 2012). This variability highlights the need for a deeper understanding of nutrient-sterol interactions to optimize microalgal cultivation for sterol production, underscoring the nuanced relationship between phosphorus supply and sterol content in microalgae (Piepho et al., 2012).

Findings on the impact of nutrient availability on sterol biosynthesis remain inconsistent and highlight species-dependent metabolic responses. While nitrogen and phosphorus levels influence sterol metabolism, their effects vary across different microalgae, necessitating further research to elucidate regulatory mechanisms and optimize cultivation strategies for enhanced sterol production in biotechnology applications.

### Salinity

Salinity markedly influences the biochemical composition of algal cells, with a particular impact on their sterol content and membrane dynamics. *Dunaliella* species, renowned for their high salinity tolerance, have been extensively studied in this context. Research has shown that in *Dunaliella tertiolecta* and *Dunaliella salina*, sterol accumulation is highest at lower salinity levels, with maximum concentrations of 8.9 and 13 mg g_dw_^-1^, respectively, observed at 0.6 M NaCl. However, as salinity increases from approximately 21 to 75 g L^-1^, total sterol content progressively declines (Francavilla et al., 2010). This reduction can be linked to plasma membrane lipid composition adjustments, a response to salinity stress. In-depth studies, such as the analysis of the plasma membrane proteome of *D. salina*, reveal that these microalgae counteract salinity stress by up-regulating key metabolic and signal transduction pathways (Katz et al., 2007). This response is considered a strategic adaptation to modulate membrane fluidity and stability under saline conditions.

In contrast, other species like *Nitzschia laevis* exhibit a significant increase by 43.8% in sterol content when exposed to elevated sodium chloride concentrations, ranging from 10 to 30 g L^−1^ (Chen et al., 2008), reaching 5.44 mg g_dw_^-1^ at 30g L^-1^. A similar trend is observed in *Nannochloropsis salina*, where sterol content rises from ~4.5 to 7.5% of total lipid with salinity increments from 22 to 58 g L^−1^ (Bartley et al., 2013). However, in *Pavlova lutheri*, sterol levels remain unaffected across varying salinity ranges (15 to 45%), suggesting a more stable membrane composition under saline fluctuations (Ahmed et al., 2015). The enhanced salinity prompts a modification in algal cell membranes to reduce the influx of Na^+^ and Cl^−^ ions by increasing membrane rigidity. This physiological adaptation is crucial for maintaining cellular integrity and function under high salt concentrations, showcasing the remarkable adaptability of microalgae in diverse environmental conditions.

### Growth stages

In exploring microalgal species for sterol production, distinct growth phases significantly influence sterol biosynthesis, as evident from various species’ analyses. For most species, the stationary phase marks a critical period for sterol accumulation. Specifically, *Phaeodactylum tricornutum* (Ballantine et al., 1979), *Nannochloropsis oculata* and *Pavlova lutheri* (Dunstan et al., 1993) exhibit substantial sterol content at the stationary phase, accounting for 0.87%, 5.3% and 5% of total lipid, respectively. Contrastingly, *Nannochloropsis oceanica* diverges from this trend, with sterol levels showing modest increases during the early growth stages, reaching 0.75% of dry weight, followed by a rise to 1.15% of dry weight in the later stages of the culture cycle (Lu et al., 2014). This late-stage sterol increase is primarily attributed to a significant rise in cholesterol content. Concurrently, sterol biosynthetic genes displayed a coordinated upregulation as the culture progressed into the late log phase. All genes studied, except DXS and SMT1, showed elevated transcriptional activity, peaking in the late log phase. These observations suggest that sterol biosynthesis and accumulation are distinctive features of late cell growth as the culture nears the stationary phase (Lu et al., 2014). A different pattern was demonstrated in *H. pluvialis* CCALA 1081, where sterol accumulation is more closely linked to high light stress than to the growth phase (Scodelaro Bilbao et al., 2016). The highest sterol levels were observed within the first three days of exposure to high light, during which sterol content increased by approximately 1,200% relative to control conditions, reaching about 2.0% of dry weight. This elevated level of phytosterols (1.8% dry weight) was sustained after six days of high-light-induced stress, suggesting that light-induced oxidative stress, rather than culture age or biomass accumulation, played a dominant role in stimulating sterol biosynthesis in *H. pluvialis*.

The diversity in timing, conditions, and quantity of sterol production across these species underlines the importance of growth stage optimization in microalgal bioengineering for enhanced sterol yield.

Various studies on microalgal sterol content reveal that environmental conditions like light intensity, temperature, nutrient availability, salinity, and growth stages significantly affect sterol synthesis, with distinct responses among species. This variation underscores the importance of species-specific cultivation approaches and highlights a substantial gap in our understanding of the underlying metabolic and genetic mechanisms. The limited range of species studied and inadequate insights into how culture conditions influence cellular sterol biosynthesis are significant hurdles to optimizing sterol production. Addressing these challenges requires broader species research and deeper exploration into their sterol metabolic pathways. Such advancements are essential for developing effective cultivation strategies, enhancing sterol yields for nutraceuticals and other applications, and emphasizing the need for targeted efforts to tap into microalgae’s biotechnological potential for sustainable sterol production.

## The potential health benefits of microalgal sterols

Sterols derived from microalgae have garnered significant interest due to their diverse biological effects and potential health benefits (**Table 4**), including anti-inflammatory, antioxidant, anti-cancer, acting in immunomodulation to reduce the impact of neurological diseases like Parkinson’s and Alzheimer’s, anti-hypercholesterolemic, and anti-diabetic (Khan et al., 2018). These compounds, integral components of cell membranes, play crucial roles in the microalgae, and when consumed by humans or animals (Lv et al., 2015).

**Table 4:** Bioactivities of sterols derived from microalgae.

Biological Activity	Microalgae Species	Major Sterols	Ref.
Cholesterol-lowering activity	*Nostoc commune*	Campesterol, β-sitosterol and clionasterol	(Rasmussen et al., 2008)
Immunomodulatory and Anti-inflammatory	*Chlorella vulgaris*	Ergosterol, 7-Dehydroporiferasterol, Ergosterol peroxide, 7-Dehydroporiferasterol peroxide, 7-oxocholesterol	(Yasukawa et al., 1996)
	*Dunaliella tertiolecta*	Ergosterol, 7-Dehydroporiferasterol	(Caroprese et al., 2012)
	*Nannochloropsis oculata*	—–	(Sanjeewa et al., 2016)
Antituberculosis	*Isochrysis galbana*	24-Oxocholesterol acetate, Ergost-5-en-3β-ol, Cholest-5-en-24-1,3-(acetyloxy)-, 3β-ol and others	(Prakash et al., 2010)
Anti-cancer	*Navicula incerta*	Stigmasterol, and 5β-Hydroxysitostanol	(Kim et al., 2014)
	*Chlorella vulgaris*	Ergosterol peroxide	(Yasukawa et al., 1996)
	*Nannochloropsis oculata*	——	(Sanjeewa et al., 2016)
Neuromodulatory	*Dunaliella tertiolecta*	Ergosterol, and 7-Dehydroporiferasterol	(Francavilla et al., 2012)
Antioxidant	*Nannochloropsis oculata*	—–	(Sanjeewa et al., 2016)
	*Dunaliella tertiolecta*	Ergosterol, and 7-Dehydroporiferasterol	(Francavilla et al., 2012)

The biological effects of microalgal sterols include cholesterol-lowering effects. Microalgal sterols are structurally similar to cholesterol and can inhibit its absorption in the human intestine. This leads to decreased blood cholesterol levels, which benefits cardiovascular health. Studies have shown that specific microalgal sterols can effectively reduce LDL (low-density lipoprotein) cholesterol, often called ‘bad’ cholesterol. The cyanobacterium *Nostoc commune* has emerged as a particularly promising source of cholesterol-reducing compounds, with its sterol profile dominated by campesterol, β-sitosterol, and clionasterol (Rasmussen et al., 2008). These sterols have been shown to inhibit the activation of sterol regulatory element binding proteins (SREBPs) in hepatic cells, a critical mechanism for regulating cholesterol homeostasis. Additionally, research by Rasmussen et al. (2008) demonstrated that lipid extracts from *Nostoc commune* significantly suppressed SREBP-regulated gene expression involved in cholesterol and fatty acid synthesis, suggesting multiple synergistic mechanisms contributing to its hypocholesterolemic effects.

In addition, some sterols from microalgae exhibit immunomodulatory and anti-inflammatory properties. These compounds can modulate the immune responses by interacting with specific cellular receptors and signaling cascades, leading to reduced expression of pro-inflammatory mediators and enhanced production of anti-inflammatory factors (Sanjeewa et al., 2016). This activity makes them potentially valuable therapeutic agents for treating chronic inflammatory disorders, autoimmune conditions, and inflammation-associated diseases.

*Chlorella vulgaris* contains several bioactive sterols, including ergosterol, 7-dehydroporiferasterol, and their oxidation products such as ergosterol peroxide, which demonstrate pronounced anti-inflammatory activity (Yasukawa et al., 1996). Mechanistic studies have revealed that these compounds suppress the production of pro-inflammatory cytokines, while inhibiting inflammatory enzymes (Yasukawa et al., 1996). Similarly, sterols isolated from *Dunaliella tertiolecta*, particularly ergosterol and 7-dehydroporiferasterol, significantly reduced inflammation markers in experimental models and modulated proliferation of peripheral blood mononuclear cells, suggesting potential applications in treating immune-mediated inflammatory conditions (Caroprese et al., 2012).

Recent research on the sterol-rich fraction extracted from the microalga *Nannochloropsis oculata* has demonstrated remarkable anti-inflammatory activity with therapeutic potential for treating inflammatory disorders and certain types of cancer, including promyelocytic leukemia (Sanjeewa et al., 2016). This fraction significantly inhibited the production of nitric oxide (NO), while downregulating the expression of pro-inflammatory genes, key molecular mechanisms implicated in chronic inflammation.

Microalgal sterols possess significant antioxidant properties that contribute to their overall therapeutic potential. These compounds effectively neutralize free radicals and reactive oxygen species (ROS), thereby protecting cellular components from oxidative damage implicated in various chronic diseases, including neurodegenerative disorders, cardiovascular diseases, and certain types of cancer. The sterol-rich fraction from *Nannochloropsis* oculata has demonstrated substantial radical scavenging activity, with the ability to neutralize DPPH (2,2-diphenyl-1-picrylhydrazyl) and ABTS (2,2’-azino-bis(3-ethylbenzothiazoline-6-sulfonic acid)) radicals at concentrations comparable to standard antioxidants (Sanjeewa et al., 2016). This antioxidant capacity was correlated with a significant reduction in intracellular ROS levels and enhanced expression of endogenous antioxidant enzymes, suggesting multifaceted mechanisms for oxidative stress mitigation. Particularly intriguing is the emerging evidence for neuroprotective effects of specific microalgal sterols. *Dunaliella tertiolecta* contains ergosterol and 7-dehydroporiferasterol, which have demonstrated neuromodulatory activities in experimental models (Francavilla et al., 2012). These compounds significantly improved behavioral parameters in animal models of neurological disorders while reducing levels of inflammatory mediators in brain tissue. The observed effects were attributed to multiple mechanisms, including inhibition of microglial activation, suppression of pro-inflammatory cytokine production, and modulation of neurotransmitter systems (Francavilla et al., 2012). Additionally, these sterols demonstrated antioxidant effects in neural tissue, protecting neurons from oxidative damage associated with neurodegenerative processes.

Research has also indicated that some sterols from microalgae may have anti-cancer properties. They can induce apoptosis (programmed cell death) in cancer cells, inhibit tumor growth, suppress angiogenesis, and modulate cell signaling pathways critical for cancer progression (Luo et al., 2015). The efficacy and specific mechanisms vary considerably depending on sterol structure and cancer type, highlighting the importance of structure-activity relationships in their therapeutic applications. The marine diatom *Navicula incerta* produces several bioactive sterols with promising anticancer activity, notably stigmasterol and 5β-hydroxysitostanol (Kim et al., 2014). Similarly, *Chlorella vulgaris* contains ergosterol peroxide, a sterol derivative with documented anticancer activity across multiple cancer cell lines (Yasukawa et al., 1996). These findings open avenues for exploring microalgae-derived sterols in cancer therapy.

Furthermore, there is growing evidence that microalgal sterols might have a role in regulating blood sugar levels, thereby offering potential benefits for managing diabetes. Their exact role and mechanism in glycemic control are still under investigation. Due to their antioxidant and anti-inflammatory properties, microalgal sterols may also benefit skin health. They can potentially aid in protecting the skin from UV radiation and reduce aging signs (Luo et al., 2015).

It is important to note that while the potential health benefits of microalgal sterols are promising, more research is needed to fully understand their biological effects, optimal dosages, and possible side effects, particularly in clinical settings.

## Molecular advances for enhanced microalgal sterol production

Recent molecular advancements are transforming microalgal sterol production, bridging gaps in our understanding at the genetic level. The comprehensive sequencing of microalgal genomes has revealed the complex architecture of sterol biosynthetic pathways, enabling researchers to develop targeted molecular strategies for enhancing sterol output. These strategies encompass sophisticated genetic and metabolic engineering techniques, including precise gene editing through CRISPR/Cas9 systems (Summons et al., 2001; Naduthodi et al., 2019), strategic overexpression of rate-limiting enzymes, and selective gene silencing to redirect metabolic flux toward desired sterols (Bashir et al., 2016; Doron et al., 2016).

Foundational studies have demonstrated the efficacy of these approaches in model systems. The overexpression of squalene synthase, a key enzyme in the mevalonate pathway, in *Chlamydomonas reinhardtii*, has shown promising results in redirecting metabolic flows toward increased sterol production (Kajikawa et al., 2015). Similarly, gene silencing through RNA interference (RNAi) offers a powerful tool for downregulating competing pathways, further enhancing sterol synthesis (Kajikawa et al., 2015). D’adamo et al. (2019) pioneered this strategy by integrating three enzymes from the plant *Lotus japonicus* into *Phaeodactylum tricornutum*. This genetic intervention led to a significant upregulation in mRNA levels and amplified the activity within the native mevalonate pathway, a crucial precursor to sterol biosynthesis. As a result, this modification not only bolstered the production of essential sterols but also facilitated the synthesis of significant triterpenoids, demonstrating the potential for engineering microalgae as versatile biofactories for diverse high-value compounds.

Despite the promising outlook, the practical application of these molecular techniques in microalgae for sterol enhancement is still emerging and faces substantial real-world bottlenecks. A critical challenge is establishing stable transformation systems across diverse microalgal strains. Unlike model organisms such as *Chlamydomonas reinhardtii* and *Phaeodactylum tricornutum*, many commercially promising species remain recalcitrant to genetic manipulation due to robust cell walls, complex ploidy, or inefficient homologous recombination mechanisms (Hlavova et al., 2015; Muñoz et al., 2019). Moreover, the stability of transgene expression often diminishes over multiple generations, undermining the long-term viability of engineered strains in industrial settings (D’adamo et al., 2019). The regulatory landscape for genetically modified microalgae further complicates commercial deployment, with uncertain approval pathways and significant regional variations in permitted applications for biotechnology products (Beacham et al., 2017).

## Challenges and future directions for microalgal sterols production

Despite their potential, several challenges hinder the commercial-scale production of microalgae-derived sterols. While certain species, such as *Pavlova lutheri*, exhibit impressive sterol concentrations under optimized conditions, most microalgal species have inherently lower sterol content compared to traditional sources (Ahmed et al., 2015), requiring careful strain selection and metabolic optimization to enhance sterol production at a commercially viable scale. Additionally, sterol biosynthesis pathways in diverse microalgal lineages are not yet fully understood. Unlike the well-characterized pathways in model organisms, the regulatory mechanisms and rate-limiting steps governing sterol production in commercially relevant species remain largely uncharacterized (Fagundes et al., 2020), making maximizing sterol production through genetic or metabolic engineering challenging.

Beyond sterol yield limitations, the diversity of sterols within microalgae species presents both an opportunity and a challenge for commercial development. This biochemical diversity could lead to the discovery of novel bioactive sterols with unique pharmaceutical properties, yet simultaneously complicates standardization and commercialization efforts since cultivation conditions can significantly alter sterol profiles (Randhir et al., 2020). Furthermore, while considerable research has characterized sterol compositions across taxonomic groups, the bioactivity and health benefits of specific microalgal sterols remain underexplored. Unlike macroalgae-derived sterols, which have been extensively studied for their antioxidant, cholesterol-lowering, and anti-inflammatory properties, microalgal sterols require further investigation to determine their functional applications and potential synergistic bioactivities in commercial formulations (Luo et al., 2015; Sanjeewa et al., 2016).

While sterol yield and bioactivity represent key biological challenges, the most significant barrier to commercializing microalgal sterols is translating laboratory success to industrial-scale production. Large-scale cultivation presents substantial logistical challenges absent from controlled laboratory environments. Open pond systems are vulnerable to contamination by competing microorganisms, seasonal fluctuations in environmental conditions, and adverse weather events that can dramatically reduce productivity (Liang et al., 2004; Day et al., 2012). Even in closed photobioreactor systems, biofilm formation progressively reduces light penetration, while temperature regulation becomes increasingly energy-intensive with scale. The fundamental requirement for precise environmental control over light intensity, temperature, pH, and nutrient availability to maintain consistent sterol profiles necessitates sophisticated monitoring and automation systems that substantially increase capital and operational costs.

Current approaches to cultivation system design present an unresolved tension between cost and control. Modern photobioreactors offer precise control but entail prohibitively high setup and maintenance expenses for sterol production alone. Conversely, more economical outdoor cultivation methods introduce significant variability in sterol yields due to fluctuating environmental conditions, making production less predictable and standardization more challenging (Ruiz et al., 2016; Hoffman et al., 2017). Furthermore, current harvesting and extraction technologies are not optimized for sterol recovery, resulting in product losses and increased processing costs. Developing efficient, sterol-specific downstream processing methods remains a significant technical hurdle that must be addressed to improve overall process economics (Gilbert-López et al., 2015).

The economic viability of microalgal sterol production depends on resolving multiple interrelated challenges across the value chain. Beyond the direct production costs, regulatory uncertainties surrounding genetically modified microalgae create significant commercial barriers. Approval pathways remain unclear in many jurisdictions, with substantial regional variations in permitted applications for biotechnology products. Additionally, microalgal sterols must compete with established plant and synthetic sources in terms of cost, consistency, and quality, creating significant market entry barriers despite potential sustainability advantages.

Nevertheless, several promising innovation pathways could substantially improve the commercial outlook. Recent advances in photobioreactor design have improved light penetration, gas exchange, and overall biomass productivity (Carvalho et al., 2006; Abdur Razzak et al., 2024; Penloglou et al., 2024), though their specific impact on sterol production economics requires further evaluation. Integration of real-time monitoring systems now enables automated adjustment of cultivation parameters while minimizing energy and resource consumption (Lee et al., 2022; Porras Reyes et al., 2024).

To realize the full potential of microalgae as sterol producers, research must address several critical knowledge gaps through integrated approaches. A fundamental limitation is our poor understanding of the functional significance of individual sterols within microalgal cells. In many commercially relevant species, the roles of specific sterols in membrane integrity, stress resilience, signaling, or ecological interactions remain largely unexplored. This mechanistic understanding would enable more rational engineering of strains for targeted sterol production and better prediction of how environmental factors influence sterol profiles. Priority should be given to elucidating the complete biosynthetic pathways responsible for microalgal sterol diversity, with particular attention to the enzymes involved in key structural modifications that determine bioactivity and commercial relevance. Mapping these pathways will not only clarify evolutionary relationships across microalgal lineages but also provide a critical foundation for bioengineering efforts to produce specific, high-value sterols.

To further reduce production costs, future efforts should focus on developing more efficient harvesting techniques, such as low-energy centrifugation, membrane filtration, and bioflocculation, which streamline processing and improve extraction efficiency (Barros et al., 2015; Matter et al., 2019; Deepa et al., 2023). Moreover, utilizing alternative nutrient sources, such as wastewater-based cultivation, presents a promising strategy to lower operational expenses while improving the sustainability of microalgal biotechnology (Do et al., 2022; Kundu et al., 2024).

## Conclusion

Microalgae represent a compelling alternative to traditional sterol sources, offering unique advantages in biochemical diversity, environmental adaptability, and sustainable cultivation. The taxonomic diversity of microalgae corresponds to remarkable variation in sterol profiles, with particular species synthesizing unique compounds, including cholesterol and ergosterol, unlike plants, positioning them as a valuable platform for pharmaceutical, nutraceutical, and functional food applications.

However, despite significant progress, critical challenges hinder the full realization of microalgae as a commercially viable sterol source. The inherently low sterol content in many species, substantial variation in composition under different cultivation conditions, and the lack of complete understanding of biosynthetic regulation present significant biological barriers. Furthermore, the functional roles of microalgal sterols in cellular physiology, stress response, bioavailability, and therapeutic efficacy are poorly understood, limiting rational design approaches for enhanced yield and compositional specificity. Scaling from laboratory conditions to industrial production also presents remarkable technical and economic barriers. Current cultivation technologies struggle to deliver consistent sterol yields while maintaining economic feasibility.

Future efforts must adopt an integrative approach that bridges systems biology, synthetic biology, and bioprocess engineering. Unraveling the full regulatory networks governing sterol metabolism through multi-omics and genome editing will be critical for mapping complete sterol biosynthetic pathways across diverse microalgae and will provide the foundation for strain improvement and metabolic optimization. Simultaneously, process engineering innovations must focus on low-cost, scalable cultivation systems, particularly those leveraging waste streams and integrating carbon capture capabilities, combined with targeted process optimization for high-yield sterol production with specified molecular profiles.

In conclusion, the potential of microalgae as a sterol production platform is confirmed, but its realization hinges on overcoming significant biological, technological, and regulatory bottlenecks through coordinated research efforts. With strategic investment in fundamental research, applied technology development, and regulatory framework establishment, microalgae can be transformed from a promising alternative into a robust, industrially scalable source for sustainable sterol production, supporting the growing demands of health, nutrition, and green chemistry sectors.
